# Could We Predict POAF With a Simple Ambulatory Oscillometry Evaluating Aortic Stiffness?

**DOI:** 10.21470/1678-9741-2023-0017

**Published:** 2023-08-07

**Authors:** Ziya Apaydin, Semi Ozturk, Ali Yasar Kilinc, Ahmet Seyfeddin Gurbuz, Halil Ibrahim Biter, Ayca Gumusdag

**Affiliations:** 1 Department of Cardiology, Haseki Training and Research Hospital, Istanbul, Turkey; 2 Department of Cardiology, Istanbul Bakirkoy Dr Sadi Konuk Training and Research Hospital, Istanbul, Turkey; 3 Department of Cardiology, Arnavutkoy State Hospital, Arnavutkoy, Istanbul, Turkey; 4 Department of Cardiology, Meram Faculty of Medicine, Necmettin Erbakan University, Konya, Turkey

**Keywords:** Aortic Stiffness, Atrial Fibrillation, Coronary Artery Bypass, Pulse Pressure, Pulse Wave Velocity

## Abstract

**Objective:**

To investigate the relationship between aortic stiffness and postoperative atrial fibrillation (POAF) in patients undergoing coronary artery bypass grafting (CABG).

**Methods:**

This study included 110 patients undergoing elective isolated CABG. Aortic stiffness was measured using a noninvasive oscillometric sphygmomanometer before surgery. Characteristics of patients with and without POAF were compared.

**Results:**

POAF developed in 32 (29.1%) patients. Patients with POAF were older (63.7±8.6 *vs.* 58.3±8.4; *P*=0.014). Chronic obstructive pulmonary disease (COPD) was more common in patients with POAF (11.5% *vs.* 37.5%; *P*=0.024), whereas the frequency of hypertension, diabetes mellitus, smoking, and previous coronary artery disease did not differ. C-reactive protein and cholesterol levels were similar between patients with and without POAF. Left atrial diameter was greater in patients with POAF (35.9±1.6 *vs.* 36.7±1.7; *P*<0.039). Peripheral (p) and central (c) systolic and diastolic blood pressures were also similar between the groups, whereas both p and c pulse pressures (PP) were greater in patients with POAF (pPP: 44.3±11.9 *vs.* 50.3±11.6; *P*=0.018, cPP: 31.4±8.1 *vs.* 36.2±8.9; *P*=0.008). Pulse wave velocity (PWV) was significantly higher in POAF (8.6+1.3 *vs.* 9.4+1.3; P=0.006). PWV, pPR and COPD were independent predictors of POAF in multivariate regression analysis. In receiver operating characteristic analysis, PWV and pPP have similar accuracy for predicting POAF (PWV, area under the curve [AUC]: 0.661, 95% confidence interval [CI] [0.547-0.775], *P*=0.009) (pPP, AUC: 0.656, 95% CI [0.542-0.769], *P*=0.012).

**Conclusion:**

COPD, PWV, and PP are predictors of POAF. PP and PWV, easily measured in office conditions, might be useful for detecting patients with a higher risk of POAF.

## INTRODUCTION

Postoperative atrial fibrillation (POAF) following coronary artery bypass grafting (CABG) was shown to be associated with long-term mortality^[1]^. Numerous attempts^[2]^, including medical and surgical therapies, have been made to prevent POAF. Despite promising results, POAF is still a significant cause of morbidity and mortality, both in hospital stays and during follow-up. Therefore, recognizing patients with relatively higher risk POAF development has crucial importance.

Aortic stiffness was reported to be associated with several cardiovascular outcomes. Brachial pulse pressure (PP) is an indirect evaluation of aortic stiffness and tends to overestimate central hemodynamic. Pulse wave velocity (PWV) is solely dependent on central vascular functions, whereas PP is affected by both vascular and ventricular functions. Therefore, PWV is accepted as the gold standard and recommended by the current guidelines^[3]^ for evaluating cardiovascular risk. Previous studies proposed PP and PWV as predictors of cardiovascular and all-cause mortality^[4,5]^. Various studies^[6,7]^ showed that PWV and PP may be related to atrial fibrillation (AF) development. We aimed to evaluate the relationship between aortic stiffness parameters and POAF in patients undergoing CABG.

## METHODS

### Study Population

This study is prospective and observational. A total of 110 patients undergoing elective isolated CABG at our institution were included consecutively. Only on-pump CABG procedures were featured. Patients presenting with an acute coronary syndrome (ACS) at index hospitalization were excluded. Patients undergoing concomitant surgeries, such as valve repair/replacement, aneurysmectomy, and emergency procedures were also excluded. Patients with a history of AF (paroxysmal, persistent, permanent) and a history of any arrhythmia implying possible AF were also not considered. The same group of cardiovascular surgeons and anaesthesiologists operated on patients using the same techniques and myocardial protection. Demographic, laboratory, and clinical variables were recorded. All patients provided written informed consent and the study protocol was approved by the local ethics committee (14567952-050/924) following the Declaration of Helsinki and Good Clinical Practice guidelines.

### Postoperative Atrial Fibrillation

Patients were routinely followed by a heart rhythm monitor during intensive care unit stay. Daily electrocardiogram (ECG) was taken in the intensive care unit, as well as in the ward. Additional ECG was obtained in case the patient had any complaints such as pain, palpitation, lightheadedness, etc. POAF was defined as an occurrence of any episode of AF lasting > 30 seconds captured on ECG or monitor.

### Aortic Pulse Wave Velocity and Augmentation Index Measurement, Mobil-O-Graph® Device

Measurements were performed at index hospitalization one to three days before CABG. Patients were asked to avoid intake of caffeinated beverages, alcoholic beverages, and other stimulants within three hours of measurements. Patients had to rest in the supine position for 10 minutes before measurement at room temperature between 08:00 and 10:00.

Aortic stiffness was measured using a non-invasive oscillometric sphygmomanometer, Mobil-O-Graph® (I.E.M. GmbH, Stolberg, Germany). PWV, augmentation index, peripheral (p), and central (c) systolic blood pressure (SBP), diastolic blood pressure (DBP), and PP were calculated by the software tool. The reliability of the Mobil-O-Graph® in estimating the PWV was demonstrated in previous studies^[8]^.

### Statistical Analysis

Statistical analysis was performed with IBM Corp. Released 2013, IBM SPSS Statistics for Windows, version 22.0, Armonk, NY: IBM Corp. and MedCalc bvba version 16 (Seoul, Korea). The normality of the data was analyzed with the Kolmogorov-Smirnov test. Continuous data were expressed as mean ± standard deviation and categorical data was expressed as percentages. Differences between patient subgroups were tested using Student’s *t*-test. Categorical variables between groups were assessed with the Chi-square test or Fisher’s exact test, whichever was suitable. Logistic regression analysis was used to identify the independent predictors of POAF. Significant variables in univariate analysis were included in multivariate analysis. Two separate models were constructed. In the first model, age and pPP were excluded due to collinearity, whereas PWV was not included in the second model. Receiver operating characteristic (ROC) curves and the area under the curve (AUC) were obtained by plotting the sensitivity against the false-positive rate (1-specificity). ROC curves were compared according to DeLong et al.^[9]^. The Youden index was used to determine the optimal cutoff values of PWV and pPP for the identification of POAF. A P-value < 0.05 was considered statistically significant.

## RESULTS

### Patients’ Characteristics

**Fig. 1:**
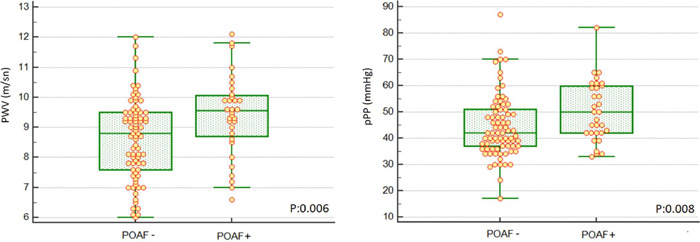
Pulse wave velocity (PWV) and peripheral pulse pressure (pPP) of patients with and without postoperative atrial fibrillation (POAF).

POAF developed in 32 (29.1%) patients. Patients with POAF were older (63.7±8.6 *vs.* 58.3±8.4; *P*=0.014). Chronic obstructive pulmonary disease (COPD) was more common in patients with POAF (11.5% *vs.* 37.5%; *P*=0.024), whereas the frequency of hypertension (HT), diabetes mellitus, smoking, and previous coronary artery disease did not differ. C-reactive protein and cholesterol levels were similar between the two groups. Left atrial diameter (LAD) was greater in patients with POAF (35.9±1.6 *vs.* 36.7±1.7 *P*<0.039). The use of medications was similar in the two groups. Baseline characteristics are presented in Table 1. p and c SBP and DBP were similar between the two groups, whereas both p and c PP were greater in patients with POAF (pPP: 44.3±11.9 *vs.* 50.3 ±11.6; *P*=0.018, cPP: 31.4±8.1 *vs.* 36.2±8.9; *P*=0.008). PWV was significantly higher in POAF (8.6±1.3 *vs.* 9.4±1.3; *P*=0.006) (Figure 1). Aortic stiffness parameters are presented in Table 1.

**Table 1:** Demographic, clinical, and laboratory characteristics of groups.

	POAF (-)	POAF (±)	P-value
	n=78	n=32	
Sex (female), n (%)	8(10.3)	3 (9.4)	> 0.999
Age (years)	59.3±8.4	63.7±8.6	0.015
Body mass index (kg/m^2^)	27.2±3.9	28.1±4.1	0.261
Smoking, n (%)	30 (38.5)	15 (46.9)	0.522
DM, n (%)	32 (41.0)	20 (39.2)	0.293
HT, n (%)	37 (47.4)	16 (50.0)	0.836
COPD, n (%)	9 (11.5)	12 (37.5)	0.024
PVD, n (%)	9 (11.5)	7 (21.9)	0.232
CAD history, n (%)	22 (28.2)	7 (21.9)	0.635
Stroke or TlA, n (%)	3 (3.8)	2 (6.3)	0.288
Ejection fraction (%)	52.3±10.7	53.4±10.1	0.639
LAD (mm)	35.9±1.6	36.7±1.7	0.039
eGFR(mL/min/1.73m^2^)	93.5±13.7	91.5±19.1	0.654
Total cholesterol (mg/dl)	268.1 ±91.6	252.6±77.7	0.192
LDL-C (mg/dl)	146.2±45.9	140.4±41.7	0.608
HDL-C (mg/dl)	39.9±7.9	39.7 ±8.0	0.944
CRP (mg/dl)	7.4±3.5	8.7±3.4	0.092
Graft count	2.9±0.9	2.9±0.8	0.957
Maximum troponin	0.1 (1.1)	0.1 (1.1)	0.609
Cross-clamping time (min)	43.9±19.2	43.2±22.0	0.870
CPB time (min)	80.8±33.4	78.6±35.9	0.760
*Medications*			
Beta-blocker, n (%)	58 (74.4)	19 (59.4)	0.119
ACE/ARB inhibitor, n (%)	35 (44.9)	9 (28.1)	0.103
Calcium channel blocker, n (%)	10(12.8)	6(18.8)	0.552
Diuretic, n (%)	22 (28.2)	8 (25)	0.732
Mineralocorticoid antagonist, n (%)	12 (15.4)	3 (9.4)	0.547
Statin, n (%)	59 (75.6)	20 (62.5)	0.164
*Aortic stiffness porometers*			
pSBP (mmHg)	132.2±18.7	139.6±16.2	0.055
pDBP (mmHg)	87.8±12.6	89.3 ±10.4	0.575
pPP (mmHg)	44.3±11.9	50.3±11.6	0.018
cSBP (mmHg)	120.8±15.9	127.2±14.6	0.054
cDBP (mmHg)	89.6±12.6	91.1±10.4	0.573
cPP (mmHg)	31.4±8.1	36.2±8.9	0.008
Alx, (%)	20.8±10.7	22.3±11.4	0.499
PWV (m/s)	8.6±1.3	9.4±1.3	0.006

Values: mean ± standard deviation; n (%); median (interquartile range)

ACE/ARB=angiotensin converting enzyme inhibitor/angiotensin receptor blocker; Aix=augmentation index; CAD=coronary artery disease; cDBP=central diastolic blood pressure; COPD=chronic obstructive pulmonary disease; CPB=cardiopulmonary bypass; cPP=central pulse pressure; CRP=C-reactive protein; cSBP=central systolic blood pressure; DM=diabetes mellitus; eGFR=estimated glomerular filtration rate; HDL-C=high density lipoprotein cholesterol; HT=hypertension; LAD=left atrial diameter; LDL-C=low density lipoprotein cholesterol;; pDBP=peripheral diastolic blood pressure; POAF=postoperative atrial fibrillation; pPP=peripheral pulse pressure; pSB-P=peripheral systolic blood pressure; PVD=peripheral vascular disease; PWV=pulse wave velocity; TIA=transient ischemic attack

### Correlations

PWV correlated strongly with age, moderately with pPP, and weakly with LAD. pPP correlated weakly with age and did not correlate with LAD (Table 2).

**Table 2:** Correlations

		Age	LAD	PWV	pPP
Age	Pearson correlation		0.214	0.856	0.241
	*P-* value		0.025	< 0.001	0.011
LAD	Pearson correlation	0.214		0.255	0.096
	*P-* value	0.025		0.007	0.323
PWV	Pearson correlation	0.856	0.255		0.514
	*P-*value	< 0.001	0.007		< 0.001
pPP	Pearson correlation	0.241	0.096	0.514	
	*P-*value	0.011	0.323	< 0.001	

LAD=left atrial diameter; pPP=peripheral pulse pressure; PWV=pulse wave velocity

### Predictors of Postoperative Atrial Fibrillation

**Fig. 2:**
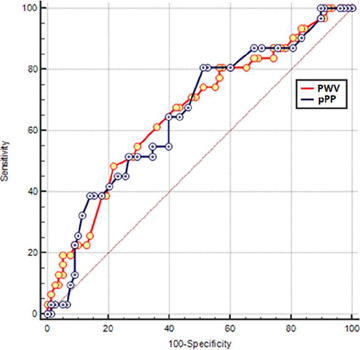
Receiver operating characteristic analysis of pulse wave velocity (PWV) and peripheral pulse pressure (pPP) for predicting postoperative atrial fibrillation.

Age, COPD, LAD, pPP, cPP, and PWV were associated with POAF in univariate logistic regression analysis (Table 3). COPD and PWV were independent predictors of POAF in the first model, whereas COPD and pPP were independent predictors in the second model (Table 4). PWV > 9.5 m/sn had 50% sensitivity and 78.2% specificity (AUC: 0.668, 95% confidence interval [CI] [0.571-0.755], *P*=0.004), whereas pPP > 41 mmHg had 80.7% sensitivity and 48.7% specificity to predict POAF (AUC: 0.656,95% CI [0.558-0.744], P=0.007]. PWV and pPP had similar accuracy for predicting POAF (difference between AUC: 0,00517; 95% CI [-0,119] - 0,129; *z* statistic: 0.0818, *P*=0.94] (Figure 2).

**Table 3:** Logistic regression analysis of postoperative atrial fibrillation.

	Univariate analysis	P-value
	OR (95% CI)	
Sex (female)	0.905 (0.224-3.655)	0.899
Age	1.067 (1.011-1.126)	0.019
Body mass index	1.063 (0.956-1.181)	0.260
Smoking	0.708 (0.309-1.626)	0.416
DM	0.614 (0.268-1.405)	0.248
HT	0.902 (0.396-2.056)	0.807
COPD	4.600 (1.697-12.471)	0.003
PVD	0.466 (0.157-1.384)	0.169
CAD history	1.403 (0.531-3.710)	0.495
Stroke or TIA	0.600 (0.095-3.773)	0.586
Ejection fraction	1.011 (0.967-1.057)	0.635
eGFR	0.992 (0.962-1.022)	0.593
Total cholesterol	0.998 (0.994-1.001)	0.248
LDL-C	0.997 (0.986-1.008)	0.604
HDL-C	0.998 (0.939-1.061)	0.943
CRP	1.105 (0.983-1.243)	0.095
LAD	1.295 (1.005-1.668)	0.045
Graft count	0.988 (0.626-1.560)	0.959
Cross-clamping time	0.998 (0.977-1020)	0.868
CPB time	0.998 (0.986-1.011)	0.758
Maximum troponin	1.021 (0.936-1.115)	0.636
Beta-blocker	1.984 (0.832-4.734)	0.122
ACE/ARB inhibitor	0.854 (0.854-5.068)	0.107
Calcium channel blocker	0.637 (0.210-1.931)	0.426
Diuretic	1.179 (0.460-3.017)	0.732
Mineralocorticoid antagonist	1.758 (0.461-6.701)	0.409
Statin	1.863 (0.771-4.505)	0.167
pSBP	1.022 (0.999-1.046)	0.060
pDBP	1.010 (0.975-1.046)	0.571
pPP	1.042 (1.006-1.080)	0.023
cSBP	1.027 (0.999-1.055)	0.058
cDBP	1.010 (0.976-1.046)	0.569
cPP	1.067 (1.015-1.122)	0.011
Alx	1.013 (0.976-1.052)	0.495
PWV	1.561 (1.119-2.177)	0.009

ACE/ARB=angiotensin converting enzyme inhibitor/angiotensin receptor blocker; Aix=augmentation index; CAD=coronary artery disease; cDBP=central diastolic blood pressure; CI=confidence interval; COPD=chronic obstructive pulmonary disease; CPB=cardiopulmonary bypass; cPP=central pulse pressure; CRP=C-reactive protein; cSBP=central systolic blood pressure; DM=diabetes mellitus; eGFR=estimated glomerular filtration rate; HDL-C=high density lipoprotein cholesterol; HT=hypertension; LAD=left atrial diameter; LDL-C=low density lipoprotein cholesterol; OR=odds ratio; pDBP=peripheral diastolic blood pressure; POAF=postoperative atrial fibrillation; pPP=peripheral pulse pressure; pSBP=peripheral systolic blood pressure; PVD=peripheral vascular disease; PWV=pulse wave velocity; TIA=transient ischemic attack

**Table 4:** Multivarate analysis of postoperative atrial fibrillation.

	First model		Second model	
	OR (95% CI)	P-value	OR (95% CI)	P-value
PWV	1.448 (1.014-2.067)	0.042	**NI**	
ppp	**NI**		1.042 (1.001-1.085)	0.046
Age	**NI**		1.038 (0.978-1.101)	0.222
COPD	4.092 (1.416-11.828)	0.009	4.997 (1.660-15.041)	0.004
LAD	1.146 (0.873-1.504)	0.327	1.158 (0.880-1.524)	0.295

CI=confidence interval; COPD=chronic obstructive pulmonary disease; LAD=left atrial diameter; NI=not included; OR=odds ratio; pPP=peripheral pulse pressure; PWV=pulse wave velocity

## DISCUSSION

POAF occurred in about one-third of patients in our study, which is compatible with the literature^[10]^. Although the frequency of POAF varies depending on the description of POAF and research methodology, it seems that POAF is still a significant cause of morbidity. This study showed for the first time that PWV and PP are associated with POAF.

The sensitivity of PP seems better than PWV despite having less specificity. Unfortunately, individual accuracies for predicting AF do not favour one over another. Nevertheless, these parameters may still aid the clinician as a fast bedside preoperative risk assessment. Historically, PP was proposed to be one of the significant determinants of cardiovascular risk^[11]^. Therefore, PP is one of the most frequently studied parameters related to vascular function, owing to the ease of measurement with a sphygmomanometer. Since PP relies on both aortic and ventricular functions, in our study we had the intention to investigate the effect of vascular function on POAF alone. Currently, PWV is the gold standard of noninvasive assessment of vascular stiffness. Initial methods, arterial catheterization, and tonometry-based methods required more time, effort, and trained staff. Thankfully, the oscillometric method simplified the process, thus PWV is measured in a few minutes and incorporated into daily routine examinations in many clinics.

Mitchell GF et al.^[12]^ showed that increased PP is a significant risk factor for new-onset AF in a large community-based sample. A previous study^[13]^ showed an association between PP and atrial volume; however, we did not find any relationship in our study. PP may be related to subclinical atrial dysfunction in patients with AF, even in patients with normal atrial size^[14]^. On the other hand, PWV in our study was related to atrial size, compatible with a previous study^[15]^.

PWV and PP are also associated with left ventricular diastolic dysfunction^[16]^, which predisposes to AF. Fumagalli S et al.^[17]^ found that vascular stiffness increasing with age is related to altered left ventricular performance, which is evaluated with longitudinal strain in the elderly with preserved ejection fraction. Therefore, subclinical left ventricular remodeling related to vascular stiffness might further cause atrial remodeling and, subsequently, AF. Furthermore, electrocardiographic studies^[18]^ demonstrated that increased P-wave dispersion is associated with altered aortic elasticity, thus increasing the risk for AF in young prehypertensive patients.

COPD is the most consistent predictor of AF and POAF in numerous studies. Despite a strong relationship, the exact pathophysiology remained to be clarified. Hypoxia and hypercapnia were speculated to cause arrhythmia^[19]^. Oxidative stress and related inflammation might be other causes triggering AF. Additionally, medications such as beta-agonists and anticholinergic drugs frequently used for COPD treatment cause AF. Pulmonary HT in COPD also might induce atrial remodeling. Additional mechanisms, including altered diastolic dysfunction and P-wave dispersion, seem to contribute to occurrences of POAF in COPD.

Central aortic hemodynamics seem more related to afterload owing to the proximity to the heart. However, we opted for pPP since it is easily measured with a simple cuff and strongly correlated with cPR Aortic stiffness is a complex measurement and is not fully understood yet. On the other hand, PWV serves as a holistic measure of aortic stiffness. Although PWV is associated with HT and age, it is less affected by other conventional risk factors^[20]^. The relationship between aortic stiffness and POAF might be due to the similarity of the remodeling process in the atria and aorta. In conclusion, PP and PWV might be useful for detecting patients with a susceptibility to POAF.

### Limitations

The small number of patients is the major limitation of this study. We excluded patients undergoing emergency, off-pump, and concomitant valve surgeries, and patients presenting with an ACS, which are daily routines of surgical practice.

## CONCLUSION

Aortic stiffness parameters of PWV and PP are associated with POAF. These easily obtained measurements should be incorporated into the risk assessment of patients undergoing CABG.

## Abbreviations, Acronyms & Symbols

ACE/ARB: Angiotensin converting enzyme inhibitor/angiotensin receptor blocker
ACS: Acute coronary syndrome
AF: Atrial fibrillation
Aix: Augmentation index
AUC: Area under the curve
c: Central
CABG: Coronary artery bypass grafting
CAD: Coronary artery disease
cDBP: Central diastolic blood pressure
CI: Confidence interval
COPD: Chronic obstructive pulmonary disease
CPB: Cardiopulmonary bypass
cPP: Central pulse pressure
CRP: C-reactive protein
cSBP: Central systolic blood pressure
DBP: Diastolic blood pressure
DM: Diabetes mellitus
ECG: Electrocardiogram
eGFR: Estimated glomerular filtration rate
HDL-C: High-density lipoprotein cholestero
HT: Hypertension
LAD: Left atrial diameter
LDL-C: Low-density lipoprotein cholesterol
NI: Not included
OR: Odds ratio
p: Peripheral
pDBP: Peripheral diastolic blood pressure
POAF: Postoperative atrial fibrillation
PP: Pulse pressure
pPP: Peripheral pulse pressure
pSBP: Peripheral systolic blood pressure
PVD: Peripheral vascular disease
PWV: Pulse wave velocity
ROC: Receiver operating characteristic
SBP: Systolic blood pressure
TIA: Transient ischemic attack
